# Five decades of natural killer cell discovery

**DOI:** 10.1084/jem.20231222

**Published:** 2024-06-06

**Authors:** Lewis L. Lanier

**Affiliations:** 1 Department of Microbiology and Immunology, University of California, San Francisco, San Francisco, CA, USA; 2 Parker Institute for Cancer Immunotherapy, University of California, San Francisco, San Francisco, CA, USA

## Abstract

The first descriptions of “non-specific” killing of tumor cells by lymphocytes were reported in 1973, and subsequently, the mediators of the activity were named “natural killer” (NK) cells by Rolf Kiessling and colleagues at the Karolinska Institute in 1975. The activity was detected in mice, rats, and humans that had no prior exposure to the tumors, major histocompatibility complex (MHC) antigen matching of the effectors and tumor cells was not required, and the cells responsible were distinct from MHC-restricted, antigen-specific T cells. In the ensuing five decades, research by many labs has extended knowledge of NK cells beyond an in vitro curiosity to demonstrate their in vivo relevance in host defense against tumors and microbial pathogens and their role in regulation of the immune system. This brief Perspective highlights a timeline of a few selected advancements in NK cell biology from a personal perspective of being involved in this quest.

## Teaser

NK cells were first described five decades ago and named in 1975. This brief Perspective highlights a timeline of a few selected advancements in NK cell biology in each decade from a personal perspective of being involved in this quest.

## Introduction

**Figure 1.**
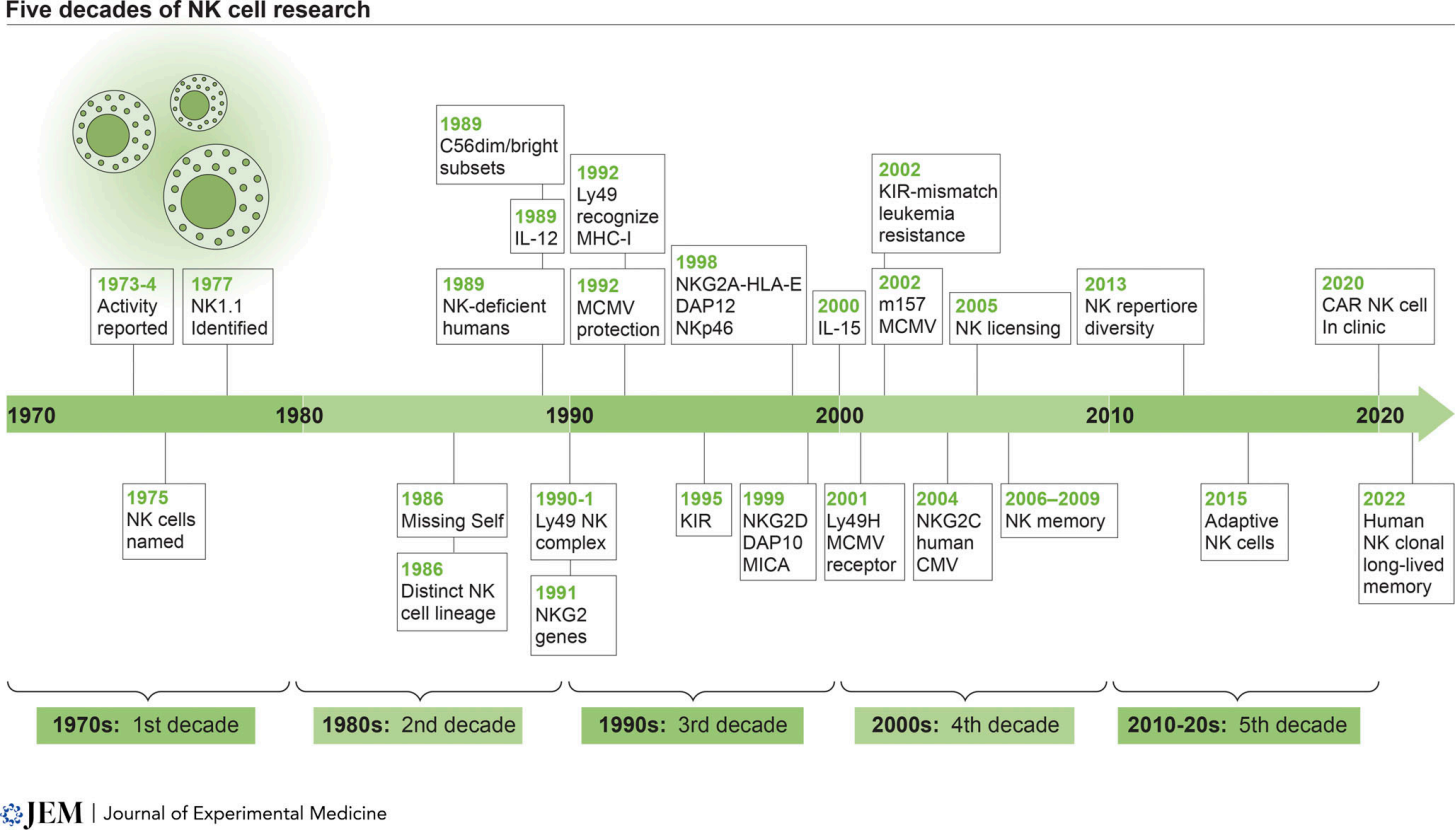
**NK cell discovery timeline.** Selected milestones in five decades of NK cell discovery. CAR, chimeric antigen receptor.

Natural killer (NK) cells were the first described member of the family of innate lymphocytes responsible for host defense against tumors and pathogens by their direct cell-mediated cytotoxic activity and secretion of cytokines and chemokines. Initially often referred to as “null cells” because of their lack of markers known to identify other lymphocytes at the time, advances in technology now enable an in-depth classification of NK cells using single-cell RNA-sequencing methods and multiparameter flow or mass cytometry to reveal the remarkable diversity within this population. Here, I present a timeline highlighting some landmark advancements in our understanding of NK cells over five decades of investigation (Fig. 1).

## First decade: 1973–1982

### Discovery of non-antigen-specific cell-mediated cytotoxicity by a new lymphocyte population

With the establishment of a simple in vitro ^51^Cr-release assay to quantitatively measure cell-mediated cytotoxicity in the late 1960s, many immunologists used this tool to investigate the ability of cytotoxic T lymphocytes to kill allogeneic cells and tumor cell targets. Using this assay, in 1974, Herberman and colleagues (Rosenberg et al., 1974) reported that peripheral blood lymphocytes from healthy individuals demonstrated the ability to kill several human lymphoblastoid cells, including in some individuals autologous lymphoblastoid cell lines. Similar observations were reported in 1975 by Jondal and Pross (Jondal and Pross, 1975; Pross and Jondal, 1975), which demonstrated “spontaneous lymphocyte-mediated cytotoxicity (SLMC) by non-thymus-derived lymphocytes from normal donors…probably caused by the complement-receptor bearing lymphocyte.”

In parallel with these studies using human lymphocytes, evidence was emerging that lymphocytes from young naïve inbred mice also possessed the ability to kill certain sensitive tumor cell lines in vitro. In 1973, Herberman and colleagues (Herberman et al., 1973) in their studies of mice immunized with Moloney sarcoma virus (MSV) to generate MSV-specific cytotoxic T cells against RBL-5 Rauscher virus-induced leukemia cells noted, “Normal lymphocytes also had low levels of cytotoxic reactivity, and this was not eliminated by anti-Thy1.” In landmark papers, Kiessling, Klein, and Wigzell also described lytic activity against syngeneic and allogeneic Moloney leukemia cells using lymphocytes from non-tumor-bearing inbred mice of several stains (Kiessling et al., 1975a, 1975b). They coined this ability to spontaneously kill tumor targets as “natural killing” and noted, “The present effector cells would seem to be lymphocytes of a new type with yet unknown biological properties” (Kiessling et al., 1975a, 1975b). In follow-up studies, Kiessling revealed that NK activity was not simply an in vitro phenomenon. This was established by demonstrating in vivo rejection of YAC-1 leukemia cells in naïve mice and by the adoptive transfer of NK cell–enriched splenocytes into mice co-administered with the leukemia cells (Kiessling et al., 1976).

Cudkowicz and Bennet in 1971 reported the rejection of parental bone marrow grafts in F1 recipient mice, a process they referred to as “hybrid resistance,” which was against the current “laws of transplantation” stating that parental tissues are tolerated by F1 recipients (Cudkowicz and Bennett, 1971). An important link of this unexplained phenomenon to NK cells was revealed in 1977 (Kiessling et al., 1977). 1977 also marked the discovery of the NK1.1 alloantigen (Glimcher et al., 1977), still the prototypic NK cell marker in C57BL/6 mice. This was followed by the establishment of a depleting monoclonal anti-NK1.1 antibody clone PK136 (Koo and Peppard, 1984), which is widely used to determine the role of NK cells in vivo.

## Second decade: 1983–1992

### The “missing-self” hypothesis and NK cells in herpesvirus defense

The second decade focused on phenotypic characterization of NK cells to allow their detection and quantitation. A morphological description of cells mediating NK cell–mediated cytotoxicity designated them as “large granular lymphocytes” based on their relatively larger size than typical lymphocytes and the presence of cytoplasmic granules later shown to contain perforin and granzymes necessary for lytic activity (Timonen et al., 1979). In 1983, the first functionally distinct subpopulations of human NK cells were identified by expression of the activating Fc receptor CD16 and CD57, a carbohydrate antigen marking the most mature NK cell population (Lanier et al., 1983). Shortly thereafter it was appreciated that both immature and mature human NK cells express CD56 (*NCAM1*) (Lanier et al., 1986c) and that the major subsets of NK cells in peripheral blood can be distinguished by the phenotypes CD56^bright^,CD16^−^ and CD56^dim^,CD16^+^ that differ in cytolytic activity and cytokine production (Nagler et al., 1989).

The earliest studies of human and mouse NK cells had clearly shown that, unlike T cells, NK cells were not “MHC restricted” as NK cells were able to lyse both syngeneic and allogeneic tumors. That NK cells preferentially kill tumor cells lacking MHC class I was demonstrated by Karre and colleagues by chemically mutagenizing the highly malignant C57BL/6 lymphoma RMA to select the H-2-negative variant RMA-S. RMA-S cells were rejected whereas the parental H-2-positive RMA tumors grew progressively (Kärre et al., 1986; Ljunggren and Kärre, 1985). These seminal studies were revolutionary and paradigm shifting. They uncovered an innate immune system mechanism to complement the function of CD8^+^ T cells to counter viruses and tumors known to downregulate MHC class I to evade T cell detection. Further, they predicted the existence of inhibitory receptors for MHC class I responsible for sparing cells expressing self-MHC class I from attack by NK cells.

Definitive evidence for NK cell–mediated killing of otherwise normal, healthy cells was provided by studies using mice with disrupted β2-microglobulin (*B2m*) genes (Höglund et al., 1991; Liao et al., 1991). NK cells from wildtype mice kill immune cells from *B2m*-deficient mice in vitro and reject syngeneic *B2m*-deficient bone marrow grafts. These experiments also provided the first evidence for “NK cell tolerance” as NK cells from the *B2m*-deficient mice were unable to attack class I–negative target cells.

A continuing debate in the ’80s was whether NK cells might be some type of unconventional T cell. With the cloning of the T cell receptor genes, it became possible to determine whether NK cells were T cells. Studies of human (Lanier et al., 1986a, 1986b) and mouse (Tutt et al., 1986) NK cells demonstrated these lymphocytes do not productively rearrange the T cell receptor genes, definitively establishing NK cells as a distinct lymphoid lineage (Lanier et al., 1986d).

Prior to 1989 there was considerable discussion about the physiological relevance of NK cells in humans. This was unambiguously resolved by Biron and colleagues, who identified the first human totally lacking NK cells yet possessing B and T lymphocytes and capable of generating antibodies (Biron et al., 1989). This patient suffered from severe herpesvirus infections and pointed the way to focus on the mechanisms by which NK cells recognize herpesviruses. Subsequent work has revealed that the cause of NK cell deficiency in this patient was due to a single loss-of-function allele of *GATA2* and *GATA2* mutations accounts for many of the patients with preferential NK cell deficiency (Mace et al., 2013). Other genetic mutations resulting in NK cell deficiencies have been described (reviewed in Mace and Orange [2016]).

The second decade was also marked by the molecular identification and cloning of genes responsible for many of the activating and inhibitory NK receptors. In mice, a cluster of genes including Ly49 (*Klra*) and NKR-P1 (*Klrb1*) was discovered on chromosome 6, designated the “NK cell gene complex” (NKC) (Yokoyama et al., 1990, 1991). Similar to the discovery of Biron that NK cells contribute to immunity against herpesviruses in humans (Biron et al., 1989), Scalzo and colleagues (Scalzo et al., 1992) reported that NK cells also protect mice from cytomegalovirus (CMV) infection. The gene responsible for CMV protection was linked to the NKC and a decade later identified by several labs as Ly49H (*Klra8*) (Brown et al., 2001; Daniels et al., 2001; Lee et al., 2001). Human NK cells were surprisingly found to express CD3ζ (Anderson et al., 1989), a key component of the T cell receptor complex in T cells. In NK cells, CD3ζ was shown to provide signal transduction for CD16, the most potent activating human NK receptor (Lanier et al., 1989). In 1991, Houchins et al. (1991) identified the human NKG2 genes encoding a family of type II C-lectin-like receptors preferentially expressed by NK cells, which were subsequently shown to include the inhibitory CD94-NKG2A and activating CD94-NKG2C receptors that recognize human HLA-E as ligands (Braud et al., 1998).

Early studies had established that IFN-γ produced by NK cells is important in immune responses to intracellular pathogens and tumors; however, what induced the production of IFN-γ by NK cells was unknown until the discovery of “NK cell stimulatory factor” by Trinchieri and colleagues (Kobayashi et al., 1989). Now designated IL-12, this cytokine, predominantly produced by myeloid cells, is essential for the production of IFN-γ by NK cells. Additionally, IL-12 is the key factor for the differentiation of naïve T cells into T helper 1 (Th1) cells and induces potent CD8^+^ T cell anti-tumor activity.

## Third decade: 1993–2002

### Inhibitory and activating NK receptors and their ligands identified

The missing-self hypothesis prompted the discovery of the inhibitory mouse Ly49 receptors (Karlhofer et al., 1992) and human killer cell immunoglobulin-like (KIR) receptors (Colonna and Samaridis, 1995; D’Andrea et al., 1995; Wagtmann et al., 1995) responsible for recognition of MHC class I—the first immune “checkpoint” receptors shown to modulate killing of tumors. The missing-self hypothesis also implied that activating receptors must exist that are countered by the inhibitory MHC class I receptors and predicted that a balance of signaling by the inhibitory and activating receptors regulates the magnitude of NK cell responses (Lanier et al., 1997).

Advances continued with the discovery of NK receptors and signaling pathways that initiate NK cell activation. The “natural cytotoxic receptors”—NKp30 (*NCR3*) (Pende et al., 1999), NKp44 (*NCR2*) (Vitale et al., 1998), and NKp46 (*NCR1*) (Pessino et al., 1998)—were identified by the Moretta group and shown to be involved in NK cell recognition of tumors. Signaling by NKp44, CD94-NKG2C, and the activating KIR and Ly49 receptors was shown to be transmitted by their non-covalent association with the immunoreceptor tyrosine-based activation motif (ITAM) –bearing DAP12 adapter protein, discovered by its structural similarity to the CD3 adapters (Lanier et al., 1998). The inhibitory Ly49, KIR, and NKG2A receptors were found to share immunoreceptor tyrosine-based inhibitory motifs in their cytoplasmic domains that recruited tyrosine phosphatases to mediate their function (Fry et al., 1996; Olcese et al., 1996; Ryan and Seaman, 1997). NKG2D, an orphan receptor discovered several years earlier (Houchins et al., 1991), was shown to require the DAP10 adapter protein for its cell surface expression and signaling (Wu et al., 1999). Ligands for NKG2D were identified in humans (MHC class I polypeptide-related sequence A, MHC class I polypeptide-related sequence B, and the UL16-binding protein) (Bauer et al., 1999; Kubin et al., 2001) and mice (Rae1, H60, and Mult1 proteins) (Carayannopoulos et al., 2002; Cerwenka et al., 2000; Diefenbach et al., 2000), which are induced by cellular stress, forming the basis of “altered-self” recognition by NK cells. NKG2D is broadly expressed on essentially all NK cells and subsets of T cells and is the focus of development of agonist and antagonist therapeutics in cancer and autoimmune diseases (Lanier, 2015). Clinical insights emerged from observations by Velardi and colleagues (Ruggeri et al., 1999), who reported that an MHC class I and KIR mismatch in the donor and recipient of hematopoietic stem cell transplants resulted in lower rates of leukemia relapse, presumably due to donor NK cells mediating a missing-self response against the tumors.

Prior studies of innate immune cells suggested that they had no antigen-specific recognition and recognized pathogens through conserved “pattern-recognition” types of receptors. In 2002, the demonstration that mouse NK cells possess an exquisitely antigen-specific receptor for a mouse CMV (MCMV) antigen countered this dogma. MCMV-encoded m157 is a glycoprotein with homology to MHC class I expressed on the surface of virally infected cells that is recognized by the activating Ly49H receptor (Arase et al., 2002; Smith et al., 2002). m157 likely initially was selected for viral immune evasion by binding with high affinity to inhibitory Ly49 receptors to suppress NK cell responses but was countered by the evolution of inhibitory Ly49 receptors into the activating Ly49H receptor (Arase et al., 2002). These findings provided a conceptual basis for the evolution of other “paired” activating and inhibitory receptors, for example, the inhibitory CD94-NKG2A and activating CD94-NKG2C receptors that recognize human CMV (Tomasec et al., 2000). Another example was the activating mouse NKR-P1C (NK.1.1) and inhibitory NKR-P1B paired receptors that compete for signaling when engaging the mouse CMV m12 protein (Aguilar et al., 2017).

## Fourth decade: 2003–2012

### NK cell “memory” and “licensing”

Classic immunology textbooks taught that innate immune cells have no memory of prior infections or antigen encounters. In 2006, this was challenged by observations of von Adrian and colleagues (O’Leary et al., 2006), who reported that mouse NK cells mediate hapten-specific contact hypersensitivity responses. T and B cells were excluded by performing the experiments in Rag-deficient mice, and adoptive transfer of the hapten-primed NK cells demonstrated an antigen-specific recall response, although to date specific NK receptors for haptens have not been identified. The ability of NK cells to mount an exquisitely antigen-specific response was validated and a molecular basis of the process was demonstrated by the ability of Ly49H^+^ NK cells to undergo clonal expansion and mount a protective response to MCMV infection when MCMV-primed memory NK cells were adoptively transferred to naïve hosts (Sun et al., 2009). Remarkably, later studies using adoptively transferred barcoded Ly49H^+^ NK cells showed that a single NK cell clone could generate 10,000 progeny at the peak of expansion after MCMV infection (Grassmann et al., 2019). Like mouse Ly49H^+^ NK cells, Lopez-Botet and colleagues (Gumá et al., 2004) revealed that human CMV infection preferentially expands NK cells expressing the CD94-NKG2C receptor, and sequencing of mitochondrial DNA (with silent mutations serving as “barcodes”) has established the long-term persistence of clonally expanded NK cells after CMV infection (Rückert et al., 2022).

Although inhibitory receptors were assumed to simply dampen NK cell activation, paradoxical findings showed that subsets of NK cells lacking a cognate ligand for self-MHC class I were less responsive than NK cells with an inhibitory Ly49 receptor for self-MHC class I (Fernandez et al., 2005; Kim et al., 2005). This has been attributed to “disarming” of NK cells lacking the inhibitory receptor or “licensing” of NK cells bearing the self-inhibiting receptor. One mechanism recently proposed to explain this event is more expression and recruitment of SHP-1 phosphatase to activating immune synapses in the “unlicensed” NK cells (lacking self-MHC class I receptors) resulting in their disarming (Schmied et al., 2023), although other models have been proposed.

## Fifth decade: 2013–today

### NK cell diversity and NK cells go to the clinic

New technologies have driven advancements in NK cell biology in the last decade. In 2013, Horowitz et al. (2013) used the newly invented mass cytometry (cytometry by time of flight) to investigate the extent of phenotypic diversity within the NK cell population in the peripheral blood of healthy adults. Using heavy metal–tagged monoclonal antibodies to stain NK cells from 12 healthy individuals, they made the remarkable discovery of 6,000–30,000 distinct phenotypic subsets in a single individual and more than 100,000 phenotypes across the donors examined—a long way from the “null” cell designation of early years. Much of this diversity can be attributed to the variegated expression of KIR genes within the NK cell population, as the KIR genes are the most polymorphic human genes (15 loci with 2,219 alleles) after MHC genes (https://www.ebi.ac.uk/ipd/kir/about/statistics/). Single-cell transcriptional analysis of NK cells has further emphasized the amazing diversity of NK cells.

The ability to characterize epigenetic changes in NK cells has revealed how mouse (Lau et al., 2018) and human (Lee et al., 2015; Schlums et al., 2015) NK cells are imprinted by CMV infection. Perhaps most remarkable are the alterations in the human memory or adaptive NK cells driven by CMV infection. In humans, these NK cells often silence by methylation their *FCERIG* and *SYK* genes, and like T cells use CD3ζ and ZAP70 for signal transduction through their ITAM-based receptors, resulting in enhanced antibody-dependent cellular cytotoxicity (Lee et al., 2015; Schlums et al., 2015). Loss of Syk and FcεR1γ does not occur in mouse Ly49H^+^ memory NK cells driven by CMV infection.

The development of methods to efficiently transduce NK cells and genetically modify them using CRISPR technology has enabled efforts to engineer NK cells for use as cellular therapeutics in cancer patients. In 2020, the first clinical use of CD19 chimeric antigen receptor NK cells in patients with lymphoid tumors showed encouraging results (Liu et al., 2020) and has prompted considerable interest in improving the efficacy of NK cell–based therapies. Challenges to be addressed are the short longevity of most NK cells and their inactivation by hostile microenvironments in solid tumors. Nonetheless, the safe experiences with clinical use of NK cells and their evolutionary evolved receptors and mechanisms for killing stressed or transformed cells provide promise for their clinical potential. Another exciting opportunity is the advancement of NK cell engagers, for example, multimeric antibodies targeting activating NK receptors and tumor-associated antigens, to the clinic (reviewed in Vivier et al. [2024]).

## Evolution of conceptual advancements on the role of NK cells in host defense and immune regulation

NK cells were initially discovered based on their ability to kill tumors and virally infected cells naturally without prior exposure. Soon thereafter it was appreciated that NK cell function was significantly augmented by type I IFNs induced during viral infections, and they served an important regulatory function by their production of IFN-γ, which activates macrophages and other immune cells and upregulates major histocompatibility II proteins for the benefit of CD4^+^ T cells (reviewed in Biron et al. [1999]). NK cells are often the first source and most abundant source of IFN-γ production in response to microbial pathogens, particularly during a first encounter with the microbe before the generation of Th1 and cytotoxic T cells that also produce IFN-γ. The search for the inducer of IFN-γ production by NK cells led to the identification of IL-12 by Trinchieri and colleagues (Chan et al., 1991), revealing a critical bridge between innate and adaptive immunity. Evidence emerged of a critical crosstalk between NK cells and other innate cells, such as dendritic cells and other myeloid populations, whereby innate cell sensing of pathogens provided the cytokines (e.g., IL-12, IL-15, and IL-18) essential for NK cell activation, cytokine production, and proliferation (reviewed in Walzer et al. [2005]). In turn, NK cells provide chemokines to attract other immune cells to the site and secrete factors, such as FLT3 ligand and IFN-γ, for maturation and activation of the dendritic cells. This interplay has proven important in the initial immune responses to microbial pathogens and tumors.

While best known for direct cytolytic function and secretion of proinflammatory cytokines, our evolving understanding of NK cells revealed their critical regulatory functions, providing important negative feedback during immune responses to prevent pathology. In the absence of NK cells, T cell responses may become too vigorous and result in collateral damage to healthy tissues. This might be achieved by direct NK cell killing of hyperactivated T cells, their elimination of dendritic cells to dampen the response, or by secretion of suppressive cytokines. Early studies revealed that IL-10–deficient mice develop colitis, and surprisingly, at the time, depletion of NK cells worsened the disease (Fort et al., 1998). The production of IL-10 by NK cells was also shown to limit pathology and CD8^+^ T cell expansion during CMV infection in mice (Lee et al., 2009). The proinflammatory versus regulatory function of NK cells is context dependent, with evidence for either a beneficial or detrimental role in autoimmune disease (reviewed in Fogel et al. [2013]).

Initial studies of NK cells focused on the cells in peripheral blood and secondary lymphoid tissues. Perhaps a first indication of tissue-resident NK cells came from studies of decidual tissues where unique populations of NK cells with distinct properties were identified (reviewed in Male and Moffett [2023]). A search for NK cells in other tissues and organs led to the discovery of innate lymphoid cells (ILCs). Many of the ILCs in tissues express “NK markers,” for example, NK1.1, NKp44, NKp46, CD161, and others, and were initially thought to be subsets of NK cells expressing IL-5, IL-22, and IL-17, as well as IFN-γ. However, subsequent work revealed that these cells comprise distinct lineages with functions akin to the Th subsets, like NK cells are the innate counterpart of CD8^+^ T cells (reviewed in Vivier et al. [2018]).

Looking forward, there are many intriguing questions to address to advance our understanding of NK cells. How do NK cells acquire antigen-specific memory? NK cells have been suggested to control T cells causing autoimmune disease—what are the mechanisms, and can this be a new therapeutic approach? How do we prevent dysfunction or re-energize NK cells in a hostile tumor microenvironment? I predict exciting and unexpected advances during the next five decades in studies of these amazing immune cells.
